# Implementation of guidelines for metabolic syndrome control in kidney transplant recipients: results at a single center

**DOI:** 10.1186/s13098-015-0083-7

**Published:** 2015-10-16

**Authors:** Inbal Houri, Keren Tzukert, Irit Mor-Yosef Levi, Michal Aharon, Aharon Bloch, Olga Gotsman, Rebecca Backenroth, Ronen Levi, Iddo Ben Dov, Dvora Rubinger, Michal Dranitzki Elhalel

**Affiliations:** Nephrology and Hypertension Services, Hadassah-Hebrew University Medical Center, 91120 Jerusalem, Israel

**Keywords:** Metabolic syndrome, Kidney transplantation, Treatment practice

## Abstract

**Background:**

Cardiovascular disease is a leading cause of death among kidney transplant recipients. Metabolic syndrome increases the risk for cardiovascular events and decreases graft survival. Lately, guidelines for management of the metabolic syndrome, primarily hypertension, diabetes mellitus (DM) and hypercholesterolemia have dramatically changed in an attempt to decrease cardiovascular risks among kidney transplant recipients. In the present study we examined whether these guideline changes had impact on our management of post-transplantation patients and the subsequent treatment outcomes for these diseases.

**Methods:**

Data were obtained from kidney transplant clinic files from two follow-up (FU) periods—between 1994–1997 and between 2008–2011. Demographic data, monitoring and screening frequency for cardiovascular risk factors, immunosuppression regimen, treatment for hypertension, diabetes and hyperlipidemia, treatment outcomes and graft function changes were compared between the two follow-up periods.

**Results:**

There was a significant increase in the percentage of patients undergoing transplantation due to renal failure secondary to diabetes and/or hypertension. Patient monitoring and screening during the second FU period were less frequent, but more targeted, reflecting changes in clinic routines. Blood pressure was better controlled in the second FU period (p < 0.01), as was hypercholesterolemia (p < 0.001). High fasting glucose levels were more prevalent among patients in the second group (p < 0.005), although more patients received treatment for DM (p < 0.001). Significantly, fewer patients experienced deterioration of kidney functions during the second FU period (p < 0.001).

**Conclusions:**

We found that guideline changes had impact on clinical practice, which translated to better control of the metabolic syndrome. DM control is challenging. Overall, stability of kidney function improved.

## Background

For kidney transplanted patients, graft failure is defined either as a need for a new renal replacement therapy or as patient’s death. Nowadays, about 50 % of graft losses are due to recipient’s death, a major cause for that being cardiovascular disease (CVD) [1, 2].

Many of the risk factors for CVD are included in the metabolic syndrome, which was first described as a combination of central obesity, dyslipidemia, hypertension and fasting hyperglycemia [3]. It has been shown that presence of metabolic syndrome in post-transplantation patients increases the risk of major adverse cardiovascular events [4, 5]. In addition, these patients have lower graft survival [6] and a higher rate of chronic transplant dysfunction [7, 8]. The defining criteria most widely accepted today in post-transplantation patients is the one proposed by the International Diabetes Federation [9, 10]. Aside from traditional risk factors for CVD, transplant-associated risk factors such as various immunosuppressive medications, chronic renal dysfunction and anemia are also acknowledged [11].

Understanding the importance of a balanced metabolic state for the general population has led to wide screening tests as well as early and aggressive interventions.

During the mid-90’s formal guidelines were not as common as they have become in the last 2 decades, more so in special populations such as kidney transplant recipients [12]. Instead, there were recommended treatment targets that were accepted as common practice. ‘Kidney Disease—Improving Global Outcomes’ (KDIGO) was established in 2003 and has since published guidelines for management of patients with kidney diseases. The 2009 KDIGO guidelines provide recommendations for the management of kidney transplant recipients, including screening and treatment goals [13].

Few large-scale studies have looked at the kidney transplant recipients population, and though most suggested significant benefit in treating hyperlipidemia, diabetes (DM) and hypertension (HTN), evidence levels are not as strong as for the general population [14]. Encouragingly, early reports describing reduced incidence of cardiovascular deaths in transplanted patients recently appeared, and suggest improvement in management of risk factors [15]. Unfortunately, treatment is still believed to be suboptimal [16, 17].

Here we aimed to elucidate the impact of changes in guidelines on our clinical management of post-transplantation patients, and to test if improvement in controlling the metabolic syndrome evolved as reflected by patients’ blood pressure, fasting glucose levels, serum cholesterol and weights. We therefore compared patient’s monitoring frequency, treatments and treatment outcomes and graft function from 2 different periods representing patients treated under two different follow-up guidelines: 1994–1997 and 2008–2011. Indeed, we found that patient monitoring has changed markedly between the two periods, more patients achieved treatments goals, and graft function was more stable during the latter follow-up period.

## Methods

### Patients and data collection

Data were obtained from files of the out-patient clinic for the follow-up (FU) of kidney transplant recipients at Hadassah Hebrew University Medical center from two time periods: 1994–1997 and 2008–2011. All patients who were under observation for the full time-period (i.e. had visits at least once a year through one of the study periods) were included. Demographic data, transplant specifics, immunosuppressive medications, patient’s weights, prevalence of screening/monitoring tests for metabolic syndrome, treatments for the different cardiovascular risk factors and the last test results were recorded at the end of the relevant FU period. As no data was available regarding patients’ height we were not able to consider BMI. Data regarding ESRD etiology was based on the diagnosis as recorded in patient file.

The outcomes examined were glucose level, cholesterol levels and blood pressure measurements as indicative for control of the metabolic syndrome diseases. We also examined changes in serum creatinine during the FU period as a representation of kidney function. The incidence of new onset diabetes mellitus was also recorded. Glucose levels, cholesterol levels and blood pressure measurements were assessed as categorical values, in order to evaluate adherence to guidelines. As only patients under observation for the whole follow up time in each period were included, no graft survival could be calculated.

With the ethic committee approval no informed consent was asked, as data were analyzed anonymously.

### Statistical analysis

In order to test the association between two categorical variables, the χ^2^ test and the Fisher’s exact test were applied. The comparison of quantitative variables between two independent groups was carried out using the *t* test or the non-parametric Mann–Whitney test when the data was not normally distributed. The logistic multivariate model was applied (using the stepwise forward method), to simultaneously assess the effect of several variables on a dependent dichotomous outcome variable. We also conducted a two-pass analysis (first considering baseline variables excluding FU period for selection into the logistic multivariable model as detailed in each section, and then testing FU period as an additional variable), receiving similar results to those achieved with the stepwise forward method. The multivariate linear regression model using the stepwise method was applied to quantitative dependent variables. All tests were two tailed and a p-value of 0.05 or less was considered statistically significant.

The following data were missing from patient records—weight was unavailable for 44 patients (15 and 29 in groups I and II respectively), pre-transplant DM was unavailable for 4 patients (3 and 1 in groups I and II respectively), last glucose was unavailable for 1 patient (from group I), last total cholesterol was unavailable for 21 patients (1 and 20 in groups I and II respectively), last LDL was unavailable for 60 patients (40 and 20 in groups I and II respectively), last TG was unavailable for 42 patients (24 and 18 in groups I and II respectively), last creatinine (and delta-creatinine) was unavailable for 6 patients (all in group I). Only available data was used for statistical analysis.

ESRD etiology was not clear for 41 patients (12 and 29 in groups I and II respectively) as they presented with ESRD. They are included in others (see Table 1) as none of them had DM, HTN or familial disease.

**Table 1:** Patient characteristics

	1994–1997	2008–2011	*p* value
Number of patients	74	238	
Sex			0.489^a^
Male	66.20 %	61.80 %	
Female	33.80 %	38.20 %	
ESRD etiology			<0.005^b^
APCKD	4.84 %	12.44 %	
Familial	11.29 %	15.79 %	
Glomerular disease	53.23 %	37.32 %	
DM	3.23 %	12.44 %	
HTN	0.00 %	5.26 %	
Other	27.42 %	16.75 %	
Creatinine at beginning of FU period (μmol/l) (mean)	134	128	0.314^c^
Weight at beginning of FU period (kg) (mean)	72.3	78	0.01^c^
Pre-transplant DM	2.8 %	16.9 %	0.002^a^
Cyclosporine treatment	90.5 %	29.0 %	<0.001^a^
Last cyclosporine level (ng/mL) (mean)	139.3	63.3	<0.0001^c^
Tacrolimus treatment	0.0 %	60.9 %	<0.001^a^
Last tacrolimus level (ng/mL) (mean)	–	5.5	
Place of transplant			<0.001^a^
Israel	80.0 %	47.8 %	
USA/Europe	6.7 %	11.4 %	
Others	13.3 %	40.8 %	
Donor living/deceased			0.002^a^
Deceased	50.8 %	29.6 %	
Living	49.2 %	70.4 %	
Donor related	31.0 %	29.9 %	0.868^a^
Age at beginning of FU (mean)	43.15 ± 11.3	49.69 ± 13.6	0.001^c^
Years on dialysis (mean)	4.13 ± 4.9	2.88 ± 3.5	0.076^c^
Age at transplantation (mean)	38.57 ± 11.8	42.29 ± 14.4	0.026^c^
Years from transplantation (mean)	4.84 ± 3.8	7.15 ± 5.0	0.001^c^

*ADPKD* adult polycystic kidney disease, *DM* diabetes mellitus, *HTN* hypertension, *FU* follow up

^a^Pearson Chi square

^b^Fisher’s exact test

^c^T-test

## Results

### Patient characteristics

312 patient files met the criteria for inclusion in the study, 74 in the first FU period and 238 in the second. Demographic and clinical characteristics are displayed in Table 1. Several significant differences between the two groups can be seen. Group II patients are older, with the mean age 47 compared with 43 in group I, in accordance with world-wide tendency to accept elderly patients to transplantation programs [1, 18, 19]. More patients in group II received kidneys from living donors (70 % compared to 50 % in group I). Additionally, etiology of ESRD differed significantly between the groups, as polycystic diseases, diabetic and hypertensive nephropathies are more prevalent in group II, while glomerular diseases were more frequent in group I, well representing the changes in ESRD etiology [1].

Patients in group II weighed more (78 kg compared to 72, p = 0.01). The prevalence of pre-transplant DM was significantly higher in group II (16.9 % compared 2.8 %, p = 0.002), in accordance with the older age as mentioned of the recipients. Notably, serum creatinine levels at the beginning of the FU period were similar between the groups.

Tacrolimus was not used in the earlier time period, while 61 % of patients in group II received this drug. Accordingly, cyclosporine use decreased drastically. Furthermore, cyclosporine mean blood levels were lower in the second group.

### Patient monitoring

**Fig. 1:**
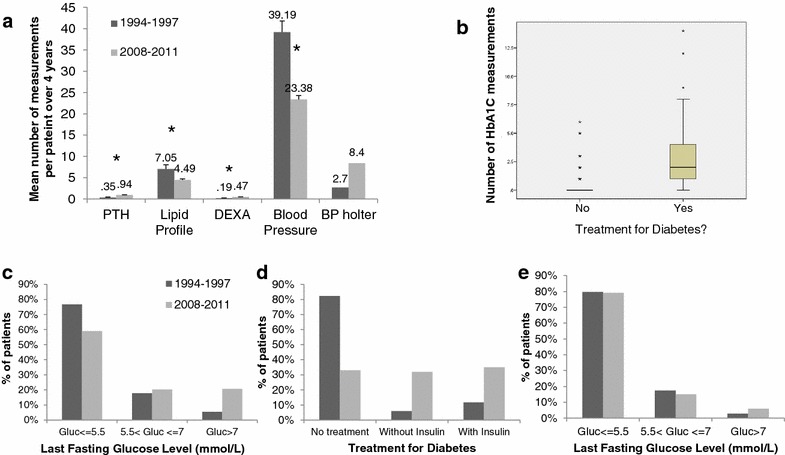
Patient monitoring frequency, treatment and outcome. **a** Average number of measurements per patient during 4 years of FU. *p < 0.05; *PTH* para-thyroid hormone, *DEXA* dual-energy X-ray absorptiometry, *BP* blood pressure. **b** Number of HbA1C measurements during the second period, comparison between patients treated for DM and patients that are not. (p < 0.001). **c** Last fasting glucose (p < 0.005). **d** DM treatment status in patients with last fasting Glucose >5.5 (p < 0.001). **e** Last fasting glucose in patients not treated for DM (p = 0.634)

As seen in Fig. 1a, frequencies of blood pressure measuring and lipid profile testing were significantly higher during the first study period. However, as blood pressure (BP) is measured at every clinic visit, number of BP measurements directly represents the number of clinic visits. Patients in group I had more clinic visits, hence more BP measurements.

24-hour BP monitoring was more common in group II, though not statistically significant. During 2008–2011 only 8.4 % of the patients underwent the test, indicating it has not yet become standard practice.

Lipid profile measurements were also more frequent in group I. We suspect that this difference is a result from a change in clinic practices, since in the second study period lipid measurements were ordered actively by the physician while in the first period they were done routinely at every clinic visit.

We were not able to compare diabetes monitoring and screening frequency. Data regarding fasting status at glucose measurement during the second study period were missing, and HbA1C measurements were not available during the early period. Patients in group II had a mean of 1.3 measurements of HbA1C during the follow-up period. Patients already being treated for diabetes had a mean of 2.85 tests compared with 0.46 in patients not treated (Fig. 1b), indicating the test is used for monitoring treatment efficiency rather than screening purposes.

Patients in group II had more PTH and DEXA measurements per 4 years of follow-up.

## Treatment outcomes

### Diabetes mellitus

The targets of DM treatment are the same for kidney transplant recipients as for all diabetic patients, and sufficiently intensive treatment should be given to maintain a HbA1C lower than 7.0–7.5 % [13], even if this means the permanent use of insulin. The recommended target was similar during the first study period [20].

The incidence of new-onset DM after transplant (NODAT) was higher in group II (14.7 % compared to 4.4 %, p = 0.024). This might reflect the higher percentage of patients receiving tacrolimus and the older age of this group.

Since HbA1C measurements were not available in the early period, we compared DM management between the patient groups by fasting glucose measurements. Glucose levels were assessed as a categorical variable, in order to differentiate controlled vs. uncontrolled diabetes (controlled ≤7 mmol/L, uncontrolled >7 mmol/L). In group I more patients had a fasting glucose <7 mmol/L (e.g. as recommended) than in group II (Fig. 1c). Factors associated in univariable analysis with a significantly greater likelihood of having glucose levels >7 mmol/L were ESRD etiology (with diabetic nephropathy, APCKD and hypertensive nephropathy increasing this likelihood, p < 0.001), treatment with tacrolimus (p = 0.03), higher weight (p < 0.001) and older age at beginning of follow-up (as a continuous variable, p < 0.001). Treatment with cyclosporine decreased this likelihood when compared to patients not treated with cyclosporine (p = 0.002). Using multivariate logistic regression model with the stepwise forward method, only ESRD etiology (p = 0.009, highest odds ratio for the diabetic nephropathy relative to familial with OR = 6.9) and higher weight (p = 0.027 with OR = 1.024) remained statistically significant. Testing FU period as an additional variable did not affect the results. Receiving treatment for DM was not included as a variable as when we tested that it reflected patients diagnosed as having DM (who naturally have higher fasting glucose levels) rather than impacting results. In conclusion, the data indicate that patients in the second FU period had a higher frequency of NODAT and higher fasting glucose levels, reflecting the higher percentage of patient undergoing transplantation because of DM and the more obese patients in the second time period.

Of patients in group II who had HbA1C measurements, 46.2 % had HbA1C >7 %. As HbA1C was tested primarily for monitoring pre-diagnosed diabetic patients, higher values were expected.

More patients with high fasting glucose levels were treated with medications through 2008-2011 than during the earlier period (Fig. 1d). Still, success of DM control was lower in group II patients, maybe reflecting the higher percentage of patients undergoing transplantation because of DM. When comparing glucose levels of patients not treated for diabetes, no significant difference was found between the two FU periods (Fig. 1e).

### Blood pressure

**Fig. 2:**
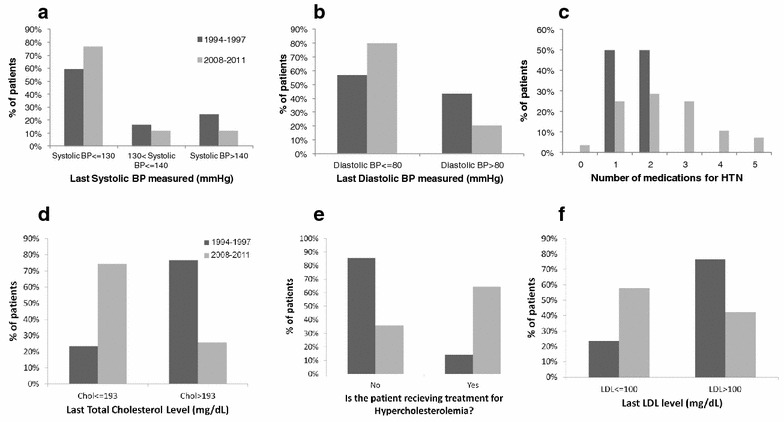
HTN and hypercholesterolemia treatment outcome. **a** Last systolic BP (p < 0.01). **b** Last diastolic BP (p < 0.001). **c** Number of medications for treatment of HTN in patients with last systolic BP >140 (p < 0.03). ** d** Last total cholesterol (mg/dL) (p < 0.001). **e** Percentage of patients receiving treatment for hypercholesterolemia among those with total cholesterol >193 (p < 0.0001). **f** Last LDL (mg/dL) (p < 0.001)

During the late 90’s, BP target was 140/90 [21], but adherence to the target was less strict. In addition, the emphasis was primarily on diastolic blood pressure, and treatment for isolated systolic hypertension was recommended only when above 160 mmHg [21]. KDIGO guidelines recommend target blood-pressure in kidney recipients of <130 mmHg systolic and <80 mmHg diastolic [13]. Blood pressure was assessed as a categorical variable, in order to differentiate controlled vs. uncontrolled hypertension (controlled ≤130/80 mmHg, uncontrolled >130/80 mmHg). Both systolic and diastolic BP were better controlled in group II (Fig. 2a, b). Factors associated in univariable analysis with higher systolic BP were ESRD etiology (with hypertensive nephropathy, glomerular disease and diabetic nephropathy APCKD increasing likelihood for higher systolic BP, p = 0.03), treatment with cyclosporine (p = 0.007), higher weight (p = 0.003), deterioration of kidney function (reflected by increase in creatinine as a continuous variable, p = 0.005) and older age at beginning of follow-up (as a continuous variable, p = 0.046). Multivariate logistic regression model using the stepwise forward method indicated only age at beginning of follow-up (p = 0.01 with OR = 1.03 per year) and increase in creatinine (p = 0.004 with OR = 1.006) as statistically significant. As patients in the second period were older, the age could not account for the better blood pressure control found in the second group. Adding treatment or no treatment for blood pressure as a variable in the multivariate analysis indicated a good correlation between having hypertension and being treated with antihypertensive drugs, but not with hypertension control.

Of patients with last systolic BP >140, in group II 42.8 % were treated with 3 or more anti-hypertensive medications, compared to no such treatment in group I (Fig. 2c). Of note, only 13.5 % of group I patients received ACEi or ARB, with an increase to 61.8 % of treated patients in group II, consistent with the increasing use of these drugs in other renal diseases. These changes might account for the better blood pressure control, however not enough patients in each group were available for such analysis.

### Hyperlipidemia

KDIGO guidelines recommend consideration of pharmacologic treatment for hypercholesterolemia at LDL above 100 mg/dL or fasting triglycerides >500 mg/dL [13]. Previous recommendations for target lipid levels were LDL <130 mg/dL or total cholesterol <200 mg/dL [22].

Due to the scarcity of LDL measurements in the earlier period we compared total cholesterol levels as well. Cholesterol levels were assessed as a categorical variable, in order to differentiate controlled vs. uncontrolled hypercholesterolemia (controlled ≤193 mg/dL, uncontrolled >193 mg/dL). Total cholesterol was better controlled at the second period (Fig. 2d). Factors associated in univariable analysis with higher cholesterol levels were ESRD etiology (with glomerular disease, familial nephropathies and diabetic nephropathy increasing said likelihood, p = 0.013), treatment with cyclosporine (p < 0.001), fewer years since transplant (p = 0.05) and surprisingly younger age at beginning of follow-up (p = 0.004). Treatment with tacrolimus decreased this likelihood (p < 0.001). However, when multivariate logistic regression model was applied using the stepwise forward method, only follow-up period remained statistically significant (p < 0.001), expressing the multitude of changes between the time periods and maybe the fact that shorter time of fasting was requested from patients before taking blood for cholesterol levels. Treatment was more common in the second period (Fig. 2e). Similar results were found for LDL levels (Fig. 2f).

58.2 % of patients in group II had TG levels under 150 compared to 40 % in group I (p < 0.02). Levels above 500 were rare (only 1 patient in group I and none in group II).

### Adherence to guidelines

Table 2 compares the overall adherence to guidelines between the two patient groups, based on relevant common practice/guideline during that period. As one can see, a significant increase in treatment targets achievement was observed for diastolic blood pressure, total cholesterol and LDL levels. No difference was found in systolic blood pressure control according to the relevant recommendation (<140 mmHg for the 1st period and then <130 mmHg for the 2nd). During the second period, fewer patients achieved serum glucose target levels then during the first period. This can be related to the higher average age and the higher prevalence of DM among patients transplanted in the second period.

**Table 2:** Overall adherence to guidelines

	Number of patients achieving target levels—1994–1997	Number of patients achieving target levels—2008–2011	p value
Systolic blood pressure	75.7 % (<140 mmHg)	76.5 % (<130 mmHg)	0.888
Diastolic blood pressure	68 % (<90 mmHg)	79.8 % (<80 mmHg)	0.029
Fasting blood glucose (<7 mmol/L)	94.5 %	79.3 %	0.005
Total cholesterol (<193 mg/dL)	23.3 %	74.3 %	<0.001
LDL	38 % (<130 mg/dL)	57.8 % (<100 mg/dL)	0.033

The percentage of patients achieving treatment goals during the two FU periods was compared. For each FU period the relevant treatment targets were used

*FU* follow up, *LDL* low density cholesterol

### Kidney functions

An important and encouraging finding is the difference noted between the groups regarding stability in kidney function. Serum creatinine levels were measured at the beginning and end of the FU period and delta-creatinine (creatinine level at the end of FU minus creatinine level at the beginning of FU) was calculated. Changes in kidney function were defined as follows: improvement = decrease in creatinine >50 μmol/l or return to normal range, deterioration = increase in creatinine 50–100 μmol/l, significant deterioration = increase in creatinine >100 μmol/L. The data show that only 5.1  % of patients in group II experienced a deterioration in kidney function during the follow-up period compared to 29.8  % in group I (Table 3). Factors associated in univariable analysis with higher delta-creatinine (reflecting deterioration in kidney function) were treatment with cyclosporine (p < 0.001), higher BP (systolic & diastolic, p = 0.005 and p = 0.01 respectively), higher cholesterol levels (p < 0.001). Treatment with tacrolimus was associated with lower delta-creatinine (p < 0.001). Weak associations were seen with age at beginning of FU (p = 0.03), age at transplant (p = 0.04) and years since transplant (p = 0.03), all associated with higher delta creatinine. Notably, no association was found between change in creatinine and ESRD etiology. When the multivariate linear regression model using the stepwise method was applied, only FU period remained significant (p < 0.001) suggesting relation between some of the factors mentioned above as the use of cyclosporine and higher blood pressure and higher levels of cholesterol. It also suggests that it is the combination of factors that results in the change

**Table 3:** Changes in kidney function

	1994–1997 (%)	2008–2011 (%)
Improvement	1.4	10.6
Stable	68.9	84.3
Deterioration	12.2%	1.7
Significant deterioration	17.6	3.4

Improvement = decrease in creatinine >50 μmol/l or return to normal range, deterioration = increase in creatinine 50–100 μmol/l, significant deterioration = increase in creatinine >100 μmol/l (p < 0.001 for all parameter tested)

## Discussion

In this study, we aimed to determine whether changes in guidelines for monitoring and treatment of CVD risk factors have been implemented in our clinic and whether treatment outcomes of these risk factors have improved. We therefore examined records from 2 different periods representing patients treated under two different follow-up guidelines, and compared monitoring frequency and treatment outcomes.

The two patient groups differed in many aspects, in accordance with trends seen in the transplantation world during the last years. Changes in patient demographics as ESRD etiology and age, increase in the percentage of living donors, emergence of new immunosuppressive drugs, subsequent change in treatment protocols and heightened awareness for the metabolic syndrome, can have an impact on the metabolic syndrome itself, and on graft function and survival, and therefore were taken into account when analyzing treatment outcomes.

Our results show that patient monitoring has changed markedly between the two periods. While some tests were done more frequently during 1994–1997 due to automatic routines, during the second study period the clinic practices have become more methodical.

DM is now the most common cause of ESRD leading to transplantation [1], is prevalent in ESRD patients as co-morbidity, and is also one of the most important risk factor for post-transplantation CVD [23–25]. The latter is also true for NODAT [26, 27]. Up-to 30 % of post-transplantation patients will develop glucose intolerance [28] that might lead to reduced survival and a higher incidence of cardiovascular events [29]. Only low percentages of patients in both periods were not treated for DM though treatment was indicated because of their fasting glucose and no difference was found between the two time periods. Fasting glucose levels and HbA1C were higher in patients from the second study period even though more patients received treatment for DM. This is mainly explained by higher mean age in group II, higher prevalence of DM as the etiology of ESRD, and the use of tacrolimus instead of cyclosporine in the second FU period.

Hypertension occurs in 60 to 80 % of renal transplant recipients, associated with an increased risk for chronic graft failure and CVD [30, 31]. Causes include donor characteristics, retained native kidneys, allograft dysfunction, immunosuppressive drugs and more [31, 32]. There is still no clearly preferred agent for treatment [10, 13, 31, 33, 34]. ACEi/ARB use was associated with a reduction in mortality though not with better graft survival [35].

We were pleased to see that BP improved dramatically between the two time periods. We believe this result from heightened physicians’ awareness and earlier and more aggressive treatment, as can be seen in the significant increase in number of medications used.

Hyperlipidemia leads to increased risk of CVD after renal transplantation [36]. In the ALERT trial the risk of major adverse cardiac event was not significantly affected by fluvastatin treatment, however patients receiving fluvastatin exhibited lower rate of cardiac death and non-fatal myocardial infarction [14]. The extension study, demonstrated a significantly reduced risk of major adverse cardiovascular events after 2 more years of FU [37]. We find highly significant lower cholesterol levels in the second study period compared to the first. Actually, the greatest difference in outcomes seen between the 2 periods appears to be in the percentage of patients who achieved target total cholesterol levels.

The significant improvement in metabolic syndrome control between the groups results from evolution in patient treatment that occurred during the last two decades. It can be attributed to an increased awareness to monitoring and treatment of the metabolic syndrome components, but it might also be the consequence of other changes. Immunosuppressive drugs, among other factors, can contribute to the metabolic syndrome in transplant patients [23]. For example, hypertension prevalence increased after treatment with calcineurin inhibitors was introduced [31]. Cyclosporine, more than tacrolimus, is associated with hypertension and hyperlipidemia [38]. Cyclosporine use decreased in the second FU period as well as mean cyclosporine blood levels. Also, patients awareness to healthy life style, including dietary habits could affect blood pressure and cholesterol levels, and this could not be evaluated.

Kidney function stability in the later follow-up period is probably multifactorial. It can be attributed, in part, to improvement in immunosuppressive regimen including the addition of tacrolimus. Another factor contributing to better graft survival might be an increased rate of living donors. However, as graft survival nowadays is highly affected by cardiovascular morbidity and mortality of the recipients, we believe that some of the change for the better in kidney function stability is related to a better control of the metabolic syndrome.

Since the study was conducted in one medical center there may be a selection bias. However, as the population in our clinic is diverse and includes many different sub-populations, we expect this selection bias to be minimal. An additional limitation was the relatively small number of patients in the first time period, which might cause selection bias. The reason for a smaller group was partly because of lower total number of patients followed at the clinic at that time. Also, small number of patients were not included because they did not meet the inclusion criteria of a yearly visit through the whole period, some of them because they went back to dialysis. This might cause a bias decreasing the differences seen between the two groups regarding the kidney functions outcome, but also regarding the blood pressure. Also, the total percentages of patients having a metabolic syndrome could not be evaluated, as no data was available about patients’ height (thus preventing us from calculating the BMI) or central obesity. Missing data was not a significant problem for data related to kidney function, glucose levels and blood pressure therefore not considered as potential source for bias. Cholesterol levels were absent for 20 out of 273 patients from the second period. As patients in this group had significantly much lower cholesterol levels, this could cause a bias by decreasing the improvements we found in controlling cholesterol levels.

In conclusion, our results indicate that changes in guidelines for monitoring and treatment goals of the metabolic syndrome have had a great impact on management of kidney transplant recipients. The majority of the patients meet the treatment goals as defined by KDIGO, even though there is still a significant number of patients who require further efforts to achieve better clinical outcomes, especially regarding DM control. The significant improvement we saw in kidney function stability over time was most gratifying, as it demonstrates that overall, the various changes in management of renal transplant patients in the last decade, including among other things modification of immunosuprressive regiments and better control of blood pressure and cholesterol levels are associated with improvement graft function.
